# Supplementary material for: The Effect of Different Colistin Dosing Regimens on Nephrotoxicity: A Cohort Study
Source: Antibiotics (Basel). 2022 Aug 5;11(8):1066. doi: 10.3390/antibiotics11081066 (PMC9405298; doi:10.3390/antibiotics11081066)
Supplement: Supplementary file 1 [file antibiotics-11-01066-s001.zip › antibiotics-1837349-supplementary.pdf]

Supplementary Table S1. Patient characteristics by treatment regimen in the full cohort.

Variable	Regimen A (n=132)	Regimen B (n=151)	Regimen C (n=23)
	n, %	n, %	n, %
**PATIENT VARIABLES**			
Gender – male	86, (65.2)	103, (68.2)	18, (78.3)
Age, years (median, IQR)	61, (48-70.5)	59, (47-68)	57, (42-74)
Weight, Kg (median, IQR)	75, (65-80)	72, (66-80)	73, (61-84)
BMI, (median, IQR)	24.8, (22.6-26.9)	25.3, (22.5-26.7)	24.5, (22-26.3)
Obesity (BMI > 30)	10, (7.6)	10, (6.7)	1, (4.3)
Ward			
Medical	28, (21.2)	39, (25.8)	17, (73.9)
Surgical	71, (53.8)	39, (25.8)	3, (13)
ICU	33, (25)	73, (48.3)	3, (13)
Charlson Comorbidity Index>3	63, (47.7)	59, (39.1)	13, (56.5)
APACHE score, (median, IQR)	10, (6-15)	12, (7-17)	14, (9-16)
Diabetes	15, (11.4)	27, (17.9)	6, (26.1)
Heart failure	10, (7.6)	20, (13.2)	2, (8.7)
Neutropenia (PMN <500/μl)	11, (8.3)	8, (5.3)	11, (47.8)
Serum creatinine at day 0, mg/dL (median, IQR)	0.64, (0.5-0.8)	0.66, (0.5-0.9)	0.88, (0.7-1)
eGFR at baseline ml/min (median, IQR)	121.8, (83.1-147.1)	116.7, (86.6-161.1)	92.9, (73.1-125.2)
Baseline eGFR > 80 ml/min	104, (78.8)	120, (80)	16, (69.6)
**INFECTION VARIABLES**			
Site of infection*			
Primary bacteraemia	14, (10.6)	33, (21.9)	8, (34.8)
UTI	7, (5.3)	11, (7.3)	1, (4.3)
Pneumonia / VAP	44, (33.3)	64, (42.4)	4, (17.4)
Abdominal infection	36, (27.3)	16, (10.6)	0, (0)
SSTI	14, (10.6)	11, (7.3)	0, (0)
Other sites	17, (12.9)	16, (10.6)	10, (43.5)
Pathogen*			
Acinetobacter spp	54, (40.9)	48, (31.8)	3, (13)
Pseudomonas spp	11, (8.3)	26, (17.2)	2, (8.7)
Klebsiella spp	30, (22.7)	39, (25.8)	1, (4.3)
Other bacteria	5, (3.8)	5, (3.3)	1, (4.3)
No bacteria isolated	32, (24.2)	33, (21.9)	16, (69.6)
Septic shock	26, (19.7)	35, (23.2)	3, (13)
Hemodynamic instability	36, (27.3)	45, (29.8)	4, (17.4)
Mechanical Ventilation	33, (25)	71, (47)	3, (13)
**TREATMENT VARIABLES**			
Empirical Treatment	33, (25)	42, (27.8)	16, (69.6)
Administration of loading dose	132, (100)	106, (70.2)	12, (52.2)
Duration of treatment, days (median, IQR)	15, (9-23)	14, (10-21)	12, (8-20)
Colistin total dose, MU (median, IQR)	135, (90-198)	135, (90-180)	108, (78-189)
Concomitant nephrotoxic agents			

Diuretics	54, (40.9)	60, (39.7)	7, (30.4)
Aminoglycosides	27, (20.5)	20, (13.2)	3, (13)
Amphotericin	9, (6.8)	3, (2)	5, (21.7)
Glycopeptides	48, (36.4)	42, (27.8)	14, (60.9)
Chemotherapy	20, (15.2)	27, (17.9)	11, (47.8)
Radiocontrast Agents	47, (35.6)	38, (25.2)	3, (13)
Non-steroidal anti-inflammatory drug	18, (13.6)	17, (11.3)	1, (4.3)
Other nephrotoxic drugs	3, (2.3)	26, (17.2)	16, (69.6)
